# Andrographolide ameliorates hepatic steatosis by suppressing FATP2-mediated fatty acid uptake in mice with nonalcoholic fatty liver disease

**DOI:** 10.1007/s11418-022-01647-w

**Published:** 2022-09-17

**Authors:** Li-Sha Ran, Ya-Zeng Wu, Yi-Wen Gan, Hong-Lian Wang, Li-Juan Wu, Chun-Mei Zheng, Yao Ming, Ran Xiong, Yong-Lin Li, Shi-Hang Lei, Xue Wang, Xiao-Qing Lao, Hong-Min Zhang, Li Wang, Chen Chen, Chang-Ying Zhao

**Affiliations:** 1 College of Integrated Chinese and Western Medicine, Southwest Medical University, Luzhou, 646000 Sichuan China; 2 Research Center for Integrative Medicine, The Affiliated Traditional Medicine Hospital of Southwest Medical University, Luzhou, 646000 Sichuan China; 3 Department of Endocrinology, The Affiliated Traditional Medicine Hospital of Southwest Medical University, Luzhou, 646000 Sichuan China; 4 School of Biomedical Sciences, University of Queensland, St Lucia, Brisbane, 4067 Australia

**Keywords:** Andrographolide, NAFLD, Hepatic steatosis, FATP2, Fatty acid uptake

## Abstract

**Abstract:**

Excessive intrahepatocellular lipid accumulation or steatosis is caused by abnormal lipid metabolism and a common character of nonalcoholic fatty liver disease (NAFLD), which may progress into cirrhosis and hepatocellular cancer. Andrographolide (Andro) is the primary active ingredient extracted from *Andrographis paniculata*, showing a protective role against dietary steatosis with the mechanism not fully understood. In this study, we showed that administration of Andro (50, 100, and 200 mg/kg/day for 8 weeks, respectively) attenuated obesity and metabolic syndrome in high-fat diet (HFD)-fed mice with improved glucose tolerance, insulin sensitivity, and reduced hyperinsulinemia, hyperglycemia, and hyperlipidemia. HFD-fed mice presented hepatic steatosis, which was significantly prevented by Andro. In vitro, Andro decreased the intracellular lipid droplets in oleic acid-treated LO2 cells. The selected RT-PCR array revealed a robust expression suppression of the fatty acid transport proteins (FATPs) by Andro treatment. Most importantly, we found that Andro consistently reduced the expression of FATP2 in both the oleic acid-treated LO2 cells and liver tissues of HFD-fed mice. Overexpression of FATP2 abolished the lipid-lowering effect of Andro in oleic acid-treated LO2 cells. Andro treatment also reduced the fatty acid uptake in oleic acid-treated LO2 cells, which was blunted by FATP2 overexpression. Collectively, our findings reveal a novel mechanism underlying the anti-steatosis effect of Andro by suppressing FATP2-mediated fatty acid uptake, suggesting the potential therapeutic application of Andro in the treatment of NAFLD.

**Graphical abstract:**

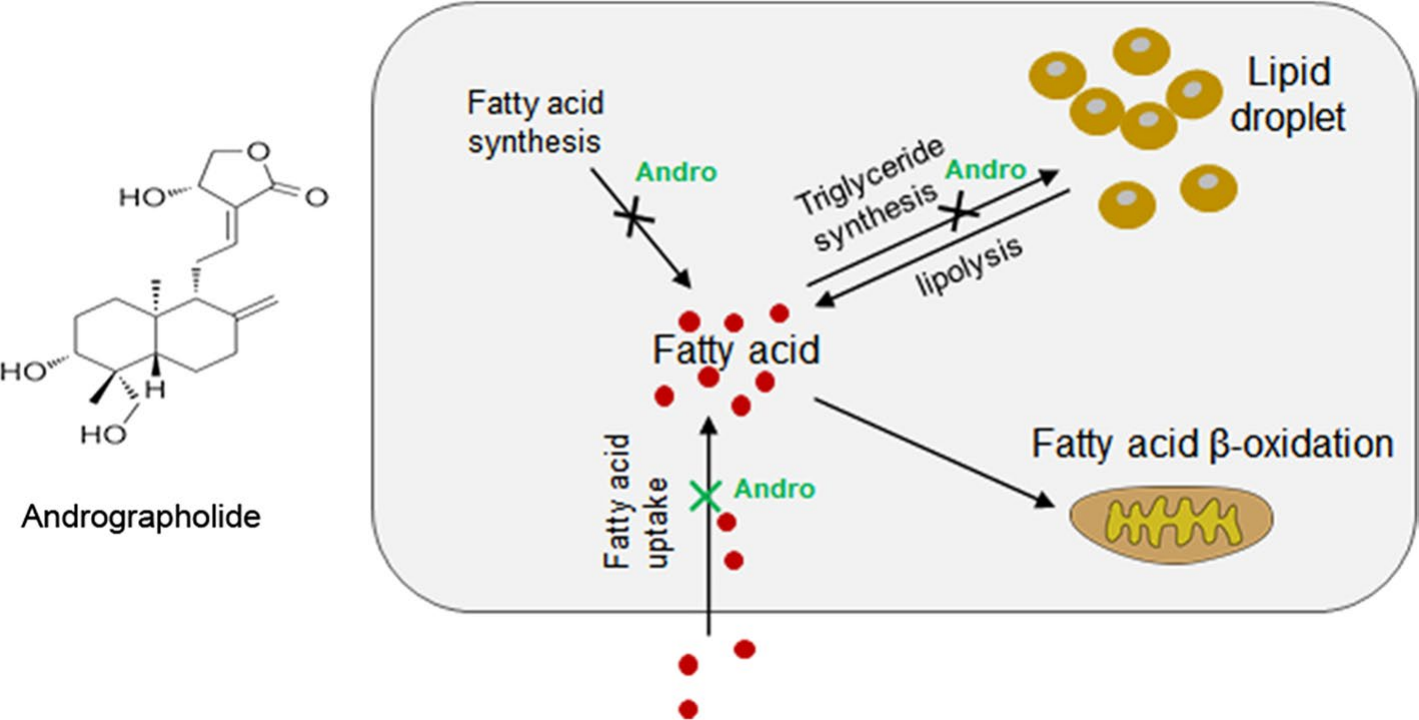

**Supplementary Information:**

The online version contains supplementary material available at 10.1007/s11418-022-01647-w.

## Introduction

Nonalcoholic fatty liver disease (NAFLD) contains a range of liver dysfunctions with the hallmark of excessive lipid accumulation in the hepatocytes or hepatic steatosis. NAFLD becomes the most common chronic liver disease, reaching about 25% of the global population [1]. The incidence of NAFLD is estimated to increase by 56% in the next 10 years in China with the epidemic of obesity [1]. NAFLD is in fact highly related to obesity-metabolic syndrome [1, 2]. Without effective treatment, NAFLD may progress to cirrhosis and liver cancer. NAFLD has become the fastest-growing cause of hepatocellular carcinoma, casting an overwhelming health burden globally [1].

The liver is an important organ for lipid metabolism. The lipid homeostasis in liver cells is controlled by lipid (fatty acid and cholesterol) import, lipogenesis (synthesis of fatty acid and triglyceride), lipid oxidation, and lipid export. Disrupting any of the above processes may cause ectopic lipid accumulation in hepatocytes and the occurrence of hepatic steatosis [3]. Hepatic steatosis itself is a considerably benign disorder but may progress to steatohepatitis, cirrhosis, and even hepatocellular carcinoma [1]. Hepatic steatosis has a complex and close relationship with insulin resistance and metabolic syndrome [1].

Andrographolide (Andro), a diterpene lactone, is one of the major bioactive ingredients of herbal medicine *Andrographis paniculata* (Burm. F.) Nees [4]. Andro presents a significant therapeutic efficacy on multiple inflammatory diseases involved with organs like lung, heart, skin, intestine, joint, and so on [5–7]. In addition, Andro also has bioactivities of anticancer, anti-oxidation, anti-bacteria, anti-human immunodeficiency virus (HIV), anti-obesity, and anti-diabetes [7–11]. In view of the liver, several lines of evidence also support a profound hepatoprotective role of Andro. Andro attenuated the histological and functional liver injury in rats challenged by carbon tetrachloride (CCl_4_) [12]. Andro also mitigated nonalcoholic steatohepatitis (NASH) by reducing liver inflammation and fibrosis in mice fed with a choline-deficient diet and in fat-loden HepG2 cells in vitro [13]. In high-fat diet (HFD)-fed mice, Andro relieved obesity and hepatic steatosis by attenuating the lipogenesis and cholesterol synthesis by regulating the sterol regulatory element-binding proteins (SREBPs) [14]. Despite these findings, the mechanism underlying the anti-steatosis effect of Andro is still not fully established. In this study, we demonstrated that Andro executes its protective role against hepatic steatosis also by suppressing fatty acid import by downregulating the fatty acid transport protein 2 (FATP2).

## Results

### Andro ameliorated metabolic syndrome in HFD-fed mice

**Fig. 1:**
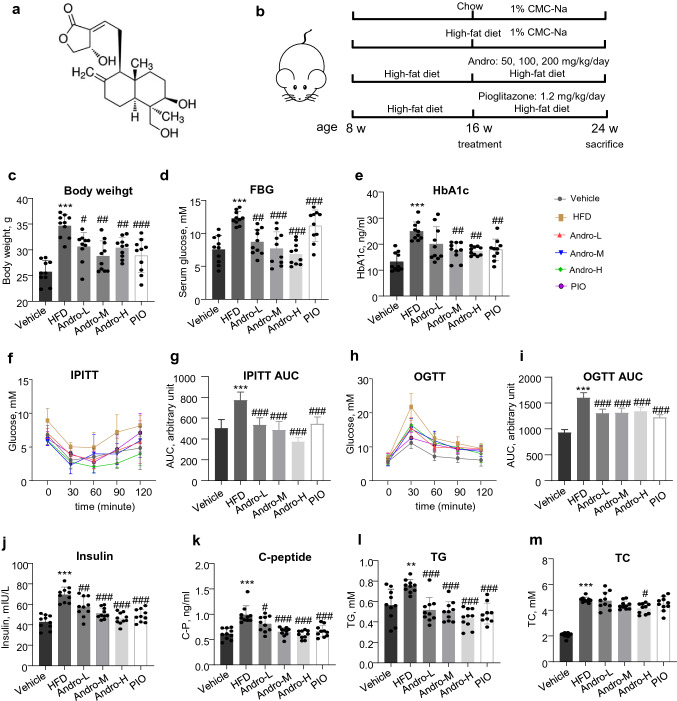
Andro treatment improved metabolic syndrome in HFD-fed mice. **a** The chemical structure of Andro. **b**. Workflow of the animal experiment. **c–e** At the end of experiment (age of week 24), body weight (**c**), fasting blood glucose (FBG, **d**), HbA1c (**e**), insulin resistance (IPITT, **f** and **g**), glucose tolerance (OGTT, **h** and **i**), serum insulin (**j**), C-peptide (**k**), total triglyceride (TG, **l**), and total cholesterol (TC, **m**) were determined. **g**, **i** are quantitation of area under curve (AUC) of (**f**, **h**), respectively. Each dot represents data from one animal. **p < 0.01 and ***p < 0.001 versus vehicle. ^#^*p* < 0.05, ^##^*p* < 0.01, and ^###^*p* < 0.001 versus HFD group

To investigate the effects of Andro on NAFLD, the C57BL/6J mice were fed with HFD for 16 weeks with different doses (50, 100, and 200 mg/kg/day) of Andro supplemented in the last 8 weeks by gavage (Fig. 1a, b). In addition, pioglitazone, an agonist of peroxisome proliferator-activated receptor α/γ (PPARα/γ) with the validated efficacy to relieve metabolic syndrome and steatosis [15], was administrated (1.2 mg/kg/day by gavage) as a positive control in a group of mice for the same period. As shown in Fig. 1c–e, the mice fed with the HFD for 16 weeks presented obvious obesity with increased levels of fasting blood glucose (FBG) and HbA1c. However, the administration of Andro significantly attenuated obesity with recovered FBG at all doses tested. A significant reduction of HbA1c was observed in mice treated with middle and high doses of Andro (Fig. 1e). Consistently, the HFD-fed mice developed significant insulin resistance and impaired glucose tolerance accompanied by hyperinsulinemia and elevated serum C-peptide, which were significantly attenuated by Andro treatment at all doses tested (Fig. 1f–k). In addition, the HFD-fed mice showed increased levels of total serum triglyceride (TG) and cholesterol (TC). Administration of Andro remarkably reduced the TG while having a slight influence on TC (Fig. 1l, m). Collectively, these data demonstrate that Andro treatment significantly improves HFD-induced metabolic syndrome. The positive drug pioglitazone showed a similar protective role to improve glucose homeostasis and hyperlipidemia.

### Andro reduced steatosis in HFD-fed mice

**Fig. 2:**
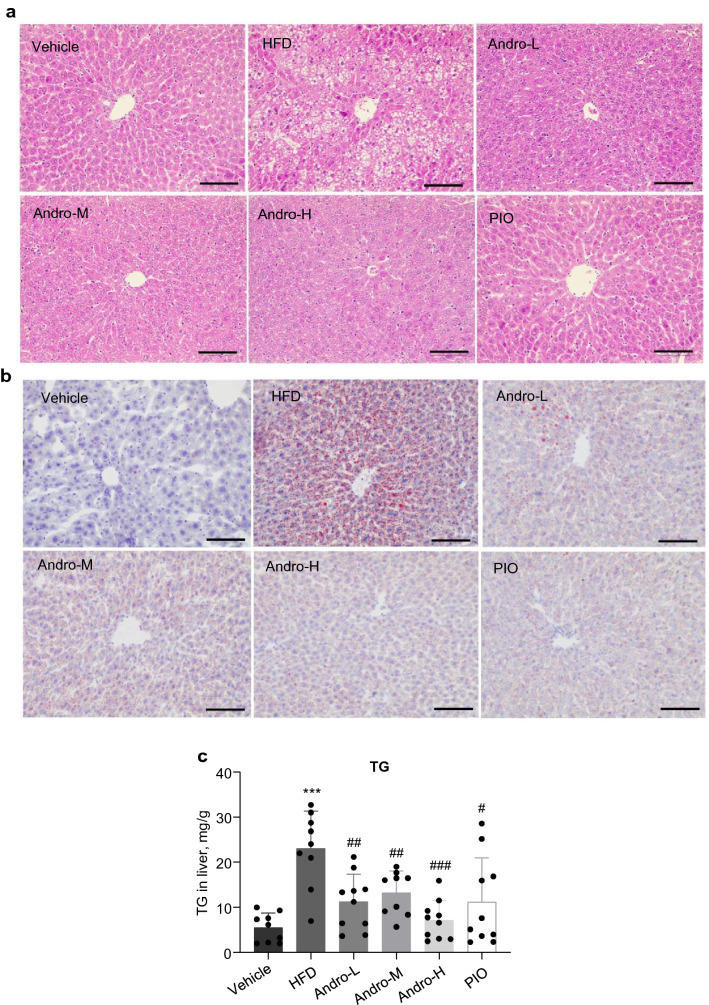
Andro treatment attenuated steatosis in HFD-fed mice. **a** Representative images of liver sections stained with HE. **b** Representative images of liver sections with Oil Red O staining to show the lipid droplets. **c** Quantitation of triglyceride in liver tissue. TG, triglyceride. Scale bar in (**a**, **b**) is 50 μm

Steatosis is a common hepatic demonstration of metabolic syndrome. We next analyzed the histological change of live tissue. By HE staining, we observed massive ballooning hepatocytes around the central vein in the liver section of HFD-fed mice, suggesting the existence of steatosis, which was further confirmed by Oil Red O staining (Fig. 2a, b). However, rare ballooning hepatocytes along with reduced staining of Oil Red O were observed in liver sections from the HFD-fed mice treated with Andro or pioglitazone. In consistence, administration of Andro also remarkably reduced the liver TG content in HFD-fed mice (Fig. 2c). Therefore, administration of Andro suppressed lipid accumulation and steatosis in HFD-fed mice.

### Andro suppressed oleic acid-induced lipid deposition in LO2 cells

**Fig. 3:**
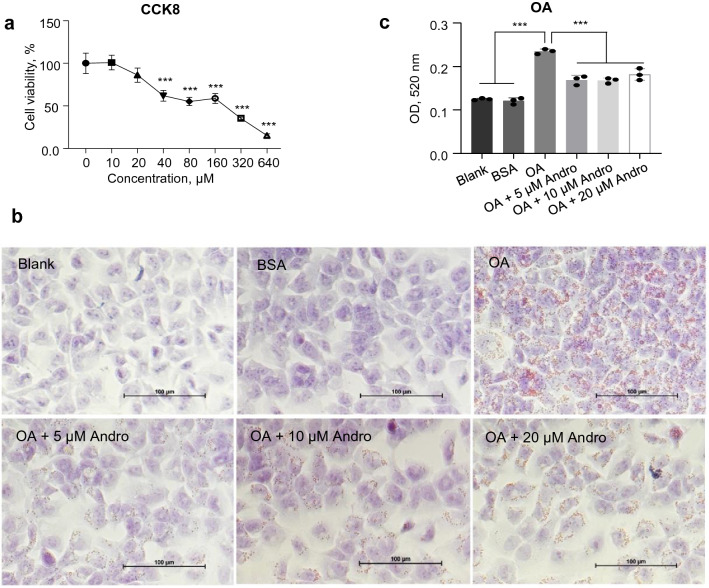
Andro suppressed the oleic acid-induced lipid accumulation in LO2 cells.** a** LO2 cells were treated with indicated concentrations of Andro for 24 h followed by the determination of viability with CCK8 reagent. ***p < 0.001 versus blank control (0). **b** LO2 cells were treated with oleic acid alone or in combination with Andro of indicated concentrations for 48 h. Intracellular lipid droplets were revealed by Oil Red O staining. Scale bar, 100 μm. **c** The Oil Red O staining was resolved by isopropanol followed by colorimetric quantitation. ***p < 0.001. OA, oleic acid

The oleic acid-treated liver cell line LO2 was then used to investigate the effect of Andro on lipid metabolism in vitro. The influence of Andro on the viability of LO2 cells was first tested by CCK8 assay. As shown in Fig. 3a, treatment with Andro of equal or less than 20 μM did not show any toxicity on LO2 cells. Therefore, the dose series of 5, 10, and 20 μM of Andro were selected for the analysis. As revealed by Oil red O staining in Fig. 3b, incubation with oleic acid (conjugated with BSA) causes massive intracellular accumulation of lipid droplets in LO2 cells compared with the non-treated (Blank) or BSA-treated cells. However, supplementation of Andro obviously suppressed the number of lipid droplets in oleic acid-treated LO2 cells at all doses tested (Fig. 3b). This was further confirmed by the colorimetric quantitation analysis of the Oil red O staining (Fig. 3c).

### Andro differentially regulated the expression of lipid metabolism genes

**Fig. 4:**
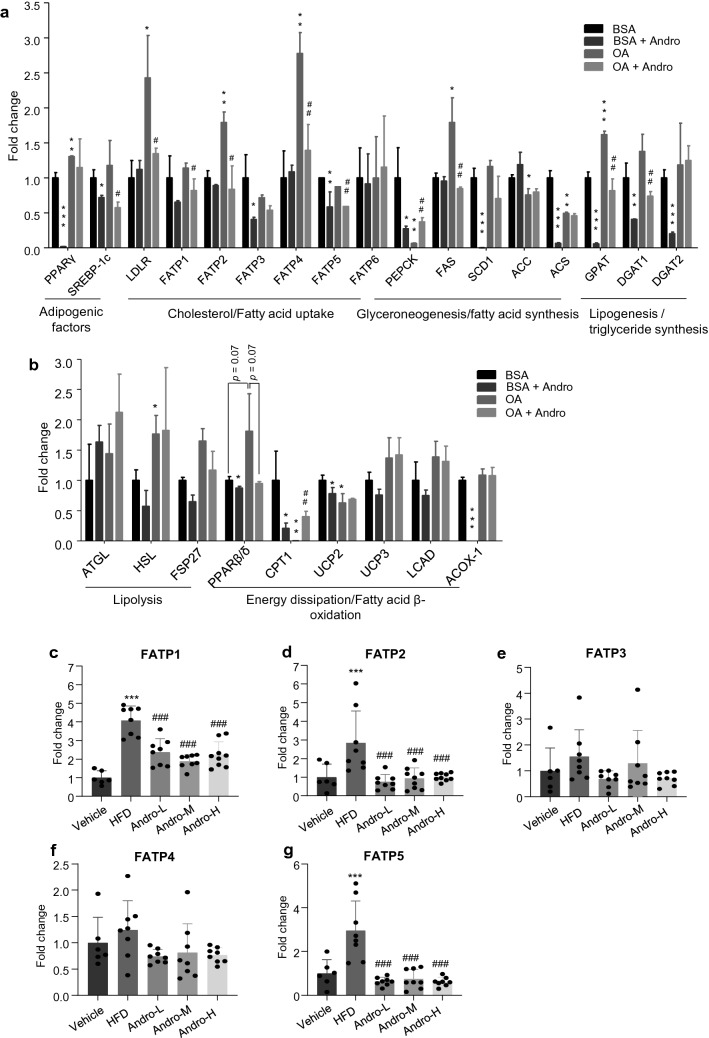
Andro differentially regulated the expression of lipid metabolism-related genes. **a**, **b** LO2 cells were treated with oleic acid or its vehicle (BSA) in combination with or without 20 μM Andro for 24 h. RT-PCR was performed to quantitate the relative expression of indicated genes associated with lipid anabolism (**a**) and catabolism (**b**), respectively. Each treatment was performed in triplicates. *p < 0.05, **p < 0.01, and ***p < 0.001 versus BSA. ^#^*p* < 0.05 and ^##^*p* < 0.01 versus OA. **c**–**g** RT-PCR to quantitate the relative expression of FATP1 to 5 in liver tissues of mice with indicated treatments. Each dot represents one animal. ***p < 0.001 versus vehicle. ^###^*p* < 0.001 versus HFD. OA, oleic acid

The intracellular homeostasis of lipid pool is synergistically regulated by the processes of lipid anabolism and catabolism. To unravel the mechanism underlying the protective role of Andro against steatosis, we analyzed the transcriptional profile of selected lipid metabolism genes in oleic acid-treated LO2 cells. As shown in Fig. 4a, in respect of lipid anabolism, Andro treatment reduced the expression of the key adipogenic factors *PPARγ* and sterol regulatory element-binding protein 1c (*SREBP-1c*) in the absence of oleic acid. *SREBP-1c* but not *PPARγ* was also decreased by Andro upon oleic acid treatment. For cholesterol/fatty acid uptake, oleic acid elevated the expression of low-density lipoprotein receptor (*LDLR*), fatty acid transport protein 2 (*FATP2*), and *FATP4*, while Andro diminished the elevations. Andro also suppressed *FATP3* and *FATP5* in LO2 cells without oleic acid treatment although both genes were not upregulated by oleic acid. *FATP5* was reduced by Andro in oleic acid treatment group. In terms of glyceroneogenesis and fatty acid synthesis, the transcription of phosphoenolpyruvate carboxykinase (*PEPCK*), stearoyl-Coenzyme A desaturase 1 (*SCD1*), and acetyl-CoA synthetase (*ACS*) were decreased by Andro treatment without oleic acid although such reduction did not occur in the presence of oleic acid. However, the expression of rate-limiting enzyme, fatty acid synthase (*FAS*), was significantly increased by oleic acid but reduced by Andro. For the process of lipogenesis or triglyceride synthesis, Andro treatment significantly decreased the expression of glycerol-3-phosphate acyltransferase (*GPAT*), diacylglycerol acyltransferase-1 (*DGAT1*), and *DGAT2* in the absence of oleic acid. Andro also significantly reduced the expression of *GPAT* and *DGAT1* in oleic acid-treated LO2 cells.

Interestingly, most of the key genes related to lipid catabolism, including lipolysis and downstream fatty acid β-oxidation, were weakly changed by Andro treatment as shown in Fig. 4b. Several genes including *PPARβ/δ*, uncoupling protein 2 (*UCP-2*), and acyl-CoA oxidase 1 (*ACOX-1*) were even reduced by Andro treatment. One exception was carnitine palmitoyltransferase 1 (*CPT1*), whose expression was reduced by Andro in the absence of oleic acid but significantly increased by Andro in the presence of oleic acid (Fig. 4b). Therefore, the transcriptional profile analysis suggested that Andro predominantly attenuates the anabolism but weakly influences the catabolism of lipids to reduce lipid accumulation in the liver cells.

### Andro treatment reduced the expression of hepatic FATPs in HFD-fed mice

It has been reported that Andro suppresses the expression of genes related to the synthesis of fatty acid and triglyceride [14]. An interesting observation in the current study is that Andro also presents a wide transcriptional suppression of the FATP gene family in oleic acid-treated LO2 cells (*FATP1*, *FATP2*, *FATP4*, and *FATP5* in Fig. 4a). Therefore, FATPs may play a role in the modulation of lipid accumulation in the hepatic cells by Andro. Next, we analyzed the expression of FATPs in liver tissues of Andro-treated mice. As shown in Fig. 4c–g, RT-PCR revealed an increased expression of *FATP1*, *FATP2*, and *FATP5* in the liver of HFD-fed mice, and the increases were again reduced by Andro. Andro also slightly reduced the expression of *FATP3* and *FATP4* in HFD-fed mice treated with the low and high dose of Andro despite no statistical significance. In contrast to the result in LO2, *FATP6* was not detected by RT-PCR in liver tissue (data not shown).

### FATP2 suppression is essential for the anti-steatosis effect of Andro

**Fig. 5:**
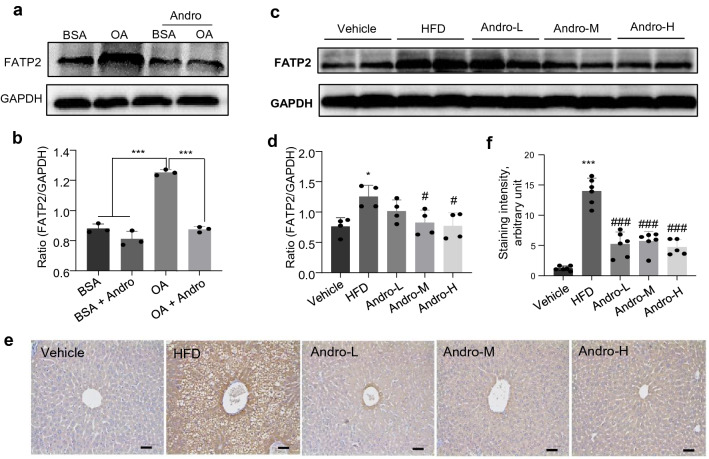
FATP2 was suppressed by Andro treatment in vivo and in vitro.** a** LO2 cells were treated with 0.5 mM BSA-conjugated OA or vehicle (BSA) in combination with or without 20 μM Andro for 48 h. The FATP2 protein level was determined by western blot. **b** Quantitation of (**a**). *n* = 3 for each treatment. ***p < 0.001. **c–f** The protein level of FATP2 in liver tissues of indicated mouse groups was checked by western blot (**c**, **d**) and immunohistochemistry (**e**, **f**). **d**, **f** Quantitation of (**c**, **e**), respectively. Each dot in (**d**, **f**) represents one animal. *p < 0.05 and ***p < 0.001 versus vehicle. ^#^*p* < 0.05 and ^###^*p* < 0.001 versus HFD. Scale bar in **e** is 50 μm. OA, oleic acid

The expression profiles of FATPs in vivo and in vitro showed that *FATP2* was the only FATP family member demonstrating consistently increased expression in both the liver of HFD-fed mice and oleic acid-treated LO2 cells. Such increased expression of *FATP2* was reduced by Andro treatment (Fig. 4). Therefore, we hypothesized that *FATP2* could be a key gene reduced by Andro to suppress fatty acid import/uptake and lipid accumulation in hepatocytes. Analysis of the protein expression by western blot showed that consistent with its mRNA expression, FATP2 was increased both in the oleic acid-treated LO2 cells and in liver tissues of HFD-fed mice, and was reduced by Andro treatment (Fig. 5a–d). Accordingly, immunostaining of FATP2 in liver sections also showed similar results (Fig. 5e, f).

**Fig. 6:**
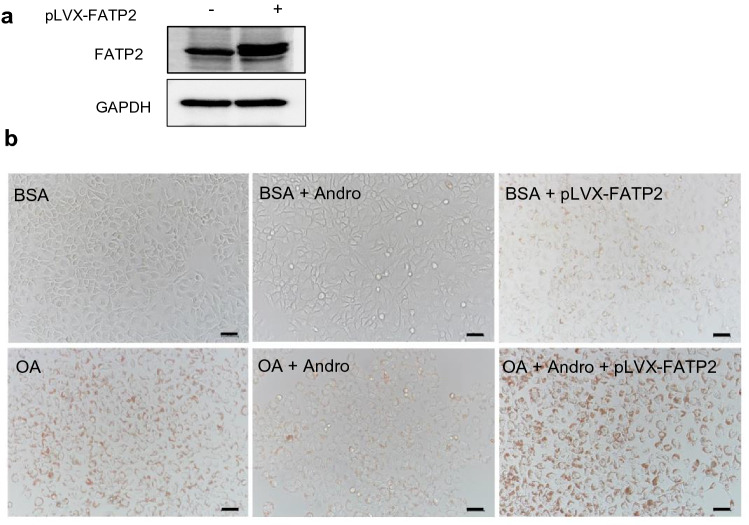
Overexpression of FATP2 abolished the anti-steatosis effect by Andro.** a** LO2 cells were transfected with pLVX-FATP2 for 48 h followed by the determination of FATP2 expression by western blot. **b** LO2 cells were transfected with or without pLVX-FATP2 followed by treatment with the indicated reagents for 48 h. Oil Red O staining was performed to show intracellular lipid droplets. Scale bar, 50 μm. OA, oleic acid

**Fig. 7:**
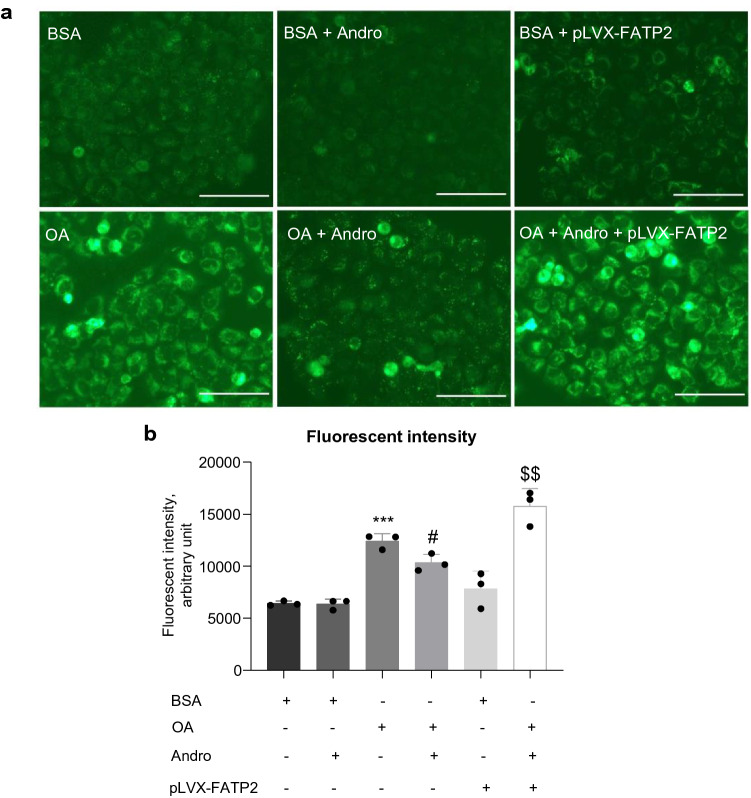
Andro suppressed oleic acid-induced fatty acid uptake which was abolished by FATP2 overexpression.** a** The LO2 cells were transfected with or without pLVX-FATP2 plasmid followed by treatment with indicated reagents. Fatty acid uptake was monitored by incubating the cells with fluorescent dye-labeled fatty acid and checked with a fluorescent microscope. Scale bar is 50 μm. **b** Flow cytometry analysis to quantitate the incorporated fluorescent dye-labeled fatty acid. Each treatment was performed in triplicates. ***p < 0.001 versus BSA group. ^#^*p* < 0.05 versus OA group. ^$$^*p* < 0.01 versus OA + Andro group. OA, oleic acid

To further confirm the functional significance of FATP2 downregulation in the protective effect of Andro against lipid accumulation in hepatocytes. FATP2 was over-expressed in LO2 cells by transfecting a vector pLVX-FATP2 harboring the CMV promoter-driven FATP2 coding sequence. Transfection of pLVX-FATP2 caused robust overexpression of FATP2 in LO2 cells as revealed by western blot (Fig. 6a). In oleic acid-treated cells, FATP2 overexpression abolished the lipid-lowering effect of Andro as demonstrated by Oil Red O staining (Fig. 7b). It is also interesting to find that FATP2 overexpression alone also resulted in a weakly increased lipid accumulation without the oleic acid induction (Fig. 7b).

### Andro reduced the fatty acid uptake in oleic acid-treated LO2 cells which was abolished by FATP2 overexpression

**Fig. 8:**
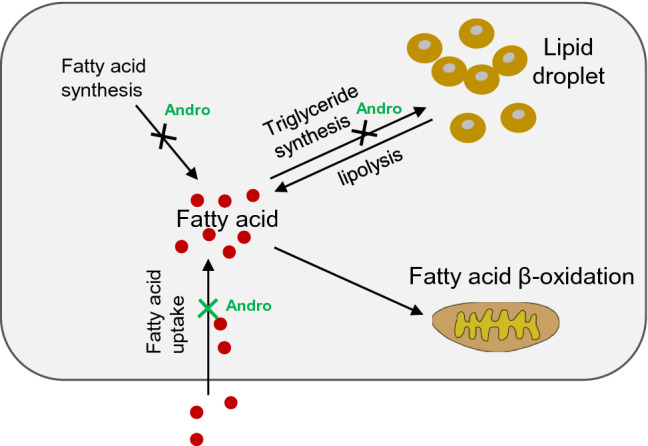
Schematic graph to summarize the anti-steatosis mechanism of Andro in hepatic cells. Andro executes its anti-steatosis effect by not only suppressing de novo fatty acid and triglyceride synthesis as previously reported (indicated by the black crosses) but also attenuating fatty acid uptake by targeting FATPs as demonstrated in this study (indicated by the green cross)

The FATPs mediate fatty acid import from the extracellular environment. Next, we performed the fluorometric fatty acid uptake assay to test whether Andro treatment could influence fatty acid uptake via regulating FATP2. The incorporated fluorescent dye-labeled fatty acid substrate was checked by the fluorescent microscope (Fig. 7a) or quantitated by flow cytometry (Fig. 7b). In the BSA-treated control cells, Andro had no significant influence on fatty acid uptake. The oleic acid-treated LO2 cells presented a significant increase in fatty acid uptake (191% of the BSA group), which was reduced by Andro treatment (85% of the oleic acid alone group). Importantly, overexpression of FATP2 by plasmid transfection completely abolished the suppression of fatty acid uptake rendered by Andro (150% of the oleic acid plus Andro group). Furthermore, overexpression of FATP2 also caused a slight increase in fatty acid uptake in BSA-treated LO2 cells. Taken together, these findings demonstrated Andro treatment reduces steatosis at least partially by suppressing FATP2-mediated fatty acid import (see Fig. 8).

## Discussion

Hepatic steatosis is the common feature of NAFLD and highly related to the occurrence and progression of metabolic syndrome, diabetes, and cardiovascular disorder [1]. In this study, we demonstrated that the natural herbal extract Andro reduced HFD-induced obesity, hyperglycemia, hyperinsulinemia, and hyperlipidemia and improved insulin resistance and glucose intolerance. Histologically, Andro treatment relieved hepatic lipid deposition or steatosis in the HFD-fed mice and in oleic acid-treated LO2 cells. These observations keep in line with the previous study and further confirm the clinical value of Andro in the treatment of metabolic syndrome-associated disorders [14, 16].

The intracellular lipid content is a combined readout orchestrated by lipid uptake, lipogenesis, lipolysis, and fatty acid oxidation. SREBP-1c is the master regulator of fatty acid synthesis [3]. In the oleic acid-treated LO2 cells in this study, Andro treatment significantly impaired the expression of SREBP-1c and FAS, the latter was the rate-limiting gene of fatty acid synthesis. On the other hand, PPARγ is the primary factor promoting adipocyte differentiation, fatty acid uptake, and triglyceride synthesis [17]. In this study, although Andro did not suppress PPARγ expression in oleic acid-treated LO2 cells, the expression of key enzymes (GPAT and DGAT1) in triglyceride synthesis was suppressed. The suppression of fatty acid and triglyceride synthesis by Andro is in line with the previous report [14].

FATP family consists of 6 members, FATP1 to 6, which are responsible for fatty acid uptake from extracellular space in various tissues [18]. FATPs show differential expression in the liver and play important roles in liver lipid metabolism [3]. The most significant and novel finding in this study was that Andro suppressed the expression of various members of FATPs in both oleic acid-treated LO2 cells and in liver tissue of HFD-fed mice. Consistently, the cellular fatty acid uptake was also impeded by Andro in vitro. Among these FATPs, we demonstrated that the suppression of FATP2 was functionally involved in the anti-steatosis effect of Andro by the gain-of-function study with overexpression of FATP2. Nevertheless, one thing worthy to note is that both FATP2 and FATP5 are abundantly expressed in the liver to facilitate fatty acid uptake and the development of hepatic steatosis [18]. Deletion or silence of either FATP2 or FATP5 reduces the liver content of triglyceride and ameliorates diet-induced steatosis in mice [19–21]. Although we only focused on FATP2 in the deep functional study in Andro-treated LO2 cells considering the extremely low expression of FATP5 (Ct value of more than 30), the potential role of FATP5 in the anti-hepatic-steatosis effect of Andro should not be excluded, as Andro treatment also robustly suppressed the expression of FATP5 in the liver tissue of HFD-fed mice (Fig. 4g). Furthermore, significant suppression of FATP1 was also observed in HFD-fed mice treated with Andro (Fig. 4c). As there is no functional study of FATP1 in liver lipid metabolism, the biological significance of such suppression by Andro in the protection of hepatic steatosis remains to clarify in the future study.

In respect of lipid catabolism, PPARβ/δ mediates the activation of fatty acid β-oxidation and promotes energy expenditure [17, 22]. In this experiment, Andro treatment suppressed PPARβ/δ expression but did not affect the expression of downstream genes involved in fatty acid β-oxidation except CPT1 in oleic acid-treated LO2 cells. Simultaneously, genes involved in lipolysis were not affected by Andro (Fig. 4b). These data suggested that Andro executed its anti-steatosis effect mainly by suppressing lipid anabolism but not catabolism. It should be emphasized that we only tested the expression of genes here without confirmation of their protein levels. In fact, it was reported that Andro promoted fatty acid β-oxidation in brown fat tissue (BAT) of HFD-fed mice [14]. Therefore, the excess calorie derived from HFD may be consumed by BAT, which may also account for the ameliorated dyslipidemia and metabolic syndrome.

## Conclusions

Collectively, this study revealed the novel activity of Andro to suppress FATP2-mediated fatty acid import, which may act synergistically with reduced fatty acid synthesis and lipogenesis, resulting in attenuated hepatic steatosis. However, how Andro regulates the expression of FATP2 in steatosis remains to address in further work. Furthermore, our results also suggest that Andro may have a broad suppression on FATP family members in steatosis although only FATP2 is selected for the functional validation in this study.

## Materials and methods

### Animal experiment

Male C57BL/6 J mice at age of 8 weeks were purchased from SPF Biotechnology Co., Ltd (Beijing, China) and were maintained in a specific pathogen-free facility with a 12/12 h light/dark cycle. The mice were randomly divided into the following 6 groups: Control, HFD, Andro-L, Andro-M, Andro-H, and PIO groups, with each group containing 10 animals. Mice in the groups of HFD, Andro-L, Andro-M, Andro-H, and PIO were given the HFD for 16 weeks while mice in the control group were treated with normal rodent chow. In the last 8 weeks, mice in the groups of Andro-L, Andro-M, and Andro-H were administrated by gavage with 50, 100, and 200 mg/kg/day Andro (dissolved in carboxymethylcellulose sodium (viscosity 800–1200, Solarbio, cat# C8621, China), respectively. Mice in Control and HFD groups were given vehicle administration by gavage. As a positive control, mice in the PIO group were given 1.2 mg/kg/day of pioglitazone by gavage. All animals had free access to food and water during the experiment. At the end of the experiment, the mice were fasted for 12 h with free access to water followed by anesthesia with pentobarbital sodium and cervical dislocation. Blood was collected for serum isolation. Liver tissue was harvested and stored at -80 °C for the isolation of RNA and protein. Part of the liver tissue was fixed in 4% neutral formaldehyde for 24 h followed by paraffin embedding for histology. All animal manipulations complied with the regulations issued and approved by the animal experimental ethics committee of Southwest Medical University (approval No. 20211103–001).

### Oral glucose tolerance test—OGTT

To determine OGTT, the mice were fasted for 12 h followed by the delivery of 2 g/kg glucose by gavage. The glucose level of tail tip blood was determined at the time points of 0, 30, 60, 90, and 120 min, respectively, by a portable glucose meter (Yuwell, China).

### Intraperitoneal insulin tolerance test—IPITT

To determine IPITT, the mice were fasted for 12 h followed by intraperitoneal injection of 0.5 U/kg insulin. The blood glucose level was monitored as above.

### Blood biochemistry

The serum levels of total triglyceride (TG) and total cholesterol (TC) were determined by the automatic biochemical analyzer (Mindray, China).

### ELISA

Serum insulin was determined with the mouse insulin ELISA kit (Zci-Bio, cat# ZC-38920, China). Serum C-peptide was checked by the mouse C-peptide ELISA kit (Zci-Bio, cat# ZC-37771, China). Blood HbA1c level was quantitated by the mouse HbA1c ESLIA kit (Zci-Bio, cat# ZC-38711, China).

### Hematoxylin and eosin (HE) Staining

The paraffin-embedded tissue was sectioned at 4 μm followed by dewaxing in xylene and rehydration in gradient ethanol. HE staining was performed with the HE Staining Kit (Beyotime, cat# C0105, China) according to the manufacturer’s instruction. The images were taken with a light microscope (Leica, ICC50W, Germany).

### Immunohistochemistry—IHC

The rehydrated liver section was subjected to microwave-mediated antigen retrieval in 10 mM citric acid (pH 6.0) for 10 min. Endogenous peroxidase activity was erased in 3% H2O2 for 15 min. After blocking with 2.5% BSA, the section was incubated with rabbit anti-FATP2 antibody (1:100, Proteintech, cat# 14,048–1-AP, USA) at 4 °C overnight. Then, the section was washed with PBS and incubated with goat anti-rabbit IgG (HRP polymer, ZSGB-Bio, cat# PV-6001, China) at RT for 20 min. After washing with PBS, chromogenesis was performed in DAB solution followed by nucleus staining with hematoxylin. After mounting in neutral balsam, the images were taken as described above.

### Preparation of bovine serum albumin (BSA)—conjugated oleic acid

To make the BSA-conjugated oleic acid, 19 μL of oleic acid (Sigma, cat# O1383, USA) was added into 3 mL of 0.1 M NaOH followed by incubation at 75 °C for 20 min. Mix the oleic acid solution with 3 mL of 20% fat-free BSA (Solarbio, cat# A88505, China) solution (in PBS) and incubate at RT for 30 min. Then, the BSA-oleic acid mixture with a final oleic acid concentration of 10 mM was filtrated through a 0.4 μm filter and stored at 4 °C in aliquots.

### Cell culture and CCK8

The LO2 cell line was obtained from the American Type Culture Collection (ATCC) and cultured in RPMI 1640 medium (Gibco, cat# 11,875,119, USA) supplemented with 10% fetal bovine serum (FBS, PAN-Biotech, cat# ST30-3302, Germany) and 1% Penicillin–Streptomycin Solution (Beyotime, cat# C0222, China) at 37 °C with 5% CO_2_ and 100% humidity. Andro was dissolved in DMSO as a stock solution of 64 mM and diluted into the indicated concentration with culture medium. For CCK8 assay, LO2 cells were seeded onto 96-well plate with 20 × 10^3^ cells each well. The next day, fresh medium containing indicated concentrations of Andro was supplemented followed by incubation for 24 h. Then, the medium was replaced with serum-free basal medium containing 10% CCK8 reagent (Dojindo, cat# CK04, China). After incubation for 4 h, the optical absorbance at wavelength of 450 nm was determined with the multifunctional microplate reader (BioTeck, Synergy 2, USA). To analyze the influence of Andro on oleic acid-induced lipid accumulation, LO2 cells were incubated with 0.5 mM BSA-conjugated oleic acid together with or without indicated concentrations of Andro for 48 h followed by Oil Red O staining or analysis of the RNA and protein expression.

### Oil red O staining

For Oil Red O staining in liver tissue, the cryostat section was performed at 10 μm for the OCT compound-embedded fresh liver tissue. The section was fixed in 4% paraformaldehyde for 10 min and washed in distilled water 2 times for 10 min each time. Then, Oil Red O staining was performed with the Modified Oil Red O Staining Kit (Beyotime, cat# C0158, China) according to the manufacturer’s instruction. For Oil Red O staining in cells, the cells were fixed in 4% paraformaldehyde for 10 min followed by washing with PBS. Oil Red O staining was performed as described above.

### RT-PCR

Total RNA was isolated from the cells or liver tissues with Trizol reagent (CWBio, cat# CW0580, China). cDNA was synthesized with the HiFiScript cDNA Synthesis Kit (CWBio, cat# CW2569, China). RT-PCR was performed with the UltraSYBR Mixture (CWBio, cat# CW0957, China). Gene expression was normalized to GAPDH. Primers used in the RT-PCR are detailed in Table S1.

### Western blot

Total protein was isolated from LO2 cells or liver tissues with RIPA lysis buffer. Protein concentration was quantitated with the BCA Protein Assay Kit (Beyotime, P0012, China). 30 μg protein was separated in 10% SDS-PAGE gel and transferred to the PVDF membrane. After blocking with 5% bovine serum albumin (BSA), the membrane was incubated with primary antibody at 4 °C overnight followed by washing with TBST. HRP-conjugated secondary antibody incubation was performed at room temperature (RT) for 1 h. The signals were developed with the ECL detection kit (Solarbio, cat# PE0010, China) and detected by the ChemiScope 600 Exp system (ClinX, China). Antibodies used in the western blot include rabbit anti-FATP2 (1:1000, Proteintech, cat# 14,048–1-AP, USA), rabbit anti-GAPDH (CST, cat# 1574, USA), and HRP-conjugated goat anti-rabbit IgG (ZSGB-Bio, cat# ZB2301, China). The gray value of the protein band was quantitated with ImageLab 6.0 software (Bio-Rad, USA).

### Plasmid construction and transfection

The murine FATP2 cDNA was amplified with primers listed in Table S1 and cloned into the pLVX vector under the human cytomegalovirus (CMV) promoter to get the overexpression plasmid pLVX-FATP2. Plasmid was prepared with the EndoFree Plasmid Mini Kit (CWBio, cat# CW2106S, China) and transfected into LO2 cells with the ViaFect™ Transfection Reagent (Promega, cat# E4981, USA).

### Fatty acid uptake assay

The fatty acid uptake ability of LO2 cells was determined by the Screen Quest™ Fluorimetric Fatty Acid Uptake Assay Kit (AAT Bioquest, cat# 36,385, USA) following the manufacturer’s instruction. Briefly, LO2 cells were seeded onto the 96-well plate with 20 × 10^3^ cells each well. The next day, the cells were transfected with or without 100 ng pLVX-FATP2 plasmid as described above. 24 h later, the cells were treated with 0.5 mM BSA-conjugated oleic acid or BSA along with or without 20 μM Andro for another 24 h. Then, the medium was replaced with serum-free medium for 1 h followed by the addition of 100 μL fluorescent fatty acid substrate for another 1 h. The cellular fluorescence intensity was monitored under a fluorescent microscope (EVOS FL Auto Cell Imaging System, Invitrogen, USA) or quantitated by flow cytometry analysis (BD Canto II, USA) with the FITC channel after trypsin digestion.

### Statistical analysis

The quantitative data were presented as mean ± SD. The SSPS 16.0 software was used for statistical analysis. One-way ANOVA followed by LSD test was applied for comparison among multiple groups. The graphs were generated in Graphpad Prism 5 software. *p* < 0.05 is regarded to be statistically significant.

## Supplementary Information

Below is the link to the electronic supplementary material.

Supplementary file1 (DOCX 18 KB)
