# Supplementary material for: Andrographolide ameliorates hepatic steatosis by suppressing FATP2-mediated fatty acid uptake in mice with nonalcoholic fatty liver disease
Source: J Nat Med. 2022 Sep 17;77(1):73–86. doi: 10.1007/s11418-022-01647-w (PMC9810587; doi:10.1007/s11418-022-01647-w)
Supplement: Supplementary file 1 — Supplementary file1 (DOCX 18 KB) [file 11418_2022_1647_MOESM1_ESM.docx]

**Table 1 Primers used in this study.**

| Primer name | Forward sequence | Reverse sequence |
| --- | --- | --- |
| Primers used in RT-PCR in LO2 cells | | |
| PPARγ | TTGTCACGGAACACGTGCAG | GAGCGGGTGAAGACTCATGT |
| SREBP-1c | CTCGTCTTCCTCTGCCTGTC | AAGGAGACGAGCACCAACAG |
| LDLR | CAGATCAACCCCCACTCGC | TTTCCTCTGCCAGCAACGTC |
| FATP1 | CCTGCCTTGATGTCCCCATT | CTGTGGGACCCTCCAGTAGA |
| FATP2 | CATTCCGGTGGAAAGGGGAA | TTGATGGAGGCCATGCCAAT |
| FATP3 | ACCCTGTCTGACCCACTGTA | GGATCAGCTCCAGCCACATT |
| FATP4 | ATCTGTAGGTGGCCCCTGAT | TTTGCACACAGGCTAGGAGG |
| FATP5 | GAGAGCTGTCGGAACGGAAG | TTACCCTCACAACCTGGCAC |
| FATP6 | TTTGGCTATGCTGGGCCTTA | ACCTCAGTGGTTGCGACATT |
| PEPCK | TTTTGGCTACAACTTCGGGC | CAGATCCAGTCTAGCACCCG |
| FAS | GCAGATACCTGGAACCACCT | AGAGGTCTTCTTGGCAATAGCA |
| SCD1 | CCCTGCTTACTTGGTGAGGG | TGCCCTAGGCTGTAGGGAAT |
| ACC1 | AGAGGGAACATCCCTACGCT | CGCTGACAAGGTGGAGTGAA |
| ACS | GCACCTGGCTTGCCTAAAAC | GTTCACTGGATGGTCAGGCA |
| GPAT | ACTGTGCTGTCCTTTGCTGT | ATTACCTGAGGGGTGGACGA |
| DGAT1 | CAAGGGCGAGTGCCAGAG | TAGGGGAGTGTGGGGAATGG |
| DGAT2 | GGTGGCTCAGCTAACCTCTC | AGAAGTGGCTTTCGCCTCTC |
| ATGL | TCCCTCCCCGTTTTTCATGG | AAGTAAGCAGGCGGTCACAT |
| HSL | GGCTCAACAGGGGAACGAAT | GGTCTGAGTTGGAGTGGTCC |
| FSP27 | CTTCTTCCTGGTGCTGGAGG | TATGGGAGAGGGACAGTGGG |
| PPARβ/ϭ | ACTGCAGCCCCCTATAGTCA | GGATCAGTTGGGTCAGTGGG |
| mCPT1 | GCCTACAGCTGAAGCCCTTA | AAGCAGGTCACACAAGTCCC |
| UCP2 | TCTGGCTTTGTCTCTAGCCG | CAACTCCACCAGCACTGAGA |
| UCP3 | TCCCTCACACAAAGTGAGCA | GAGGTGAAAGGGCCAAGCTA |
| LCAD | TCGGCTACCATTTGCTCCAA | GTCCTAGGGGTTTCAAGGCA |
| Acox-1 | TTCGAGCAAGTGAGGCACAT | AAGCACAGAGCCAAGTGTCA |
| GAPDH | CTGGCCAAGGTCATCCATGACA | TAGAGGCAGGGATGATGTTCTG |
| Primers used in RT-PCR in mouse liver sample | | |
| FATP1 | TCTTCTGCCCCAGGTGGATA | CGGGCATGGACTCTCTCATC |
| FATP2 | CCTCCTGATGATCGACCGTG | AGGCACGCCATACACATTCA |
| FATP3 | AGTTCCTGGAGTCCCTGGAG | GCTCATCCACTTGGTCTGCT |
| FATP4 | GGGGCCAATAAACTCTGCCT | TCCCAAGGGCTAAGCGAAAG |
| FATP5 | GATGCTTTAGAGCGGCAAGC | AACTTGGCCAACCCCAGAAA |
| GAPDH | CCAGCTACTCGCGGCTTTA | GTTCACACCGACCTTCACCA |
| Primers used for the construction of FATP2 overexpression vector | | |
| cDNA.h.FATP2 | GACGATGACAAGGAACTTTCCGCCATCT ACACAGTCCTG | GGTAGAATTATCTAGCCTGGGAATATTCA GAGTTTCAGGGT |
